# Selective toxicity of TGF-alpha-PE40 to EGFR-positive cell lines: selective protection of low EGFR-expressing cell lines by EGF.

**DOI:** 10.1038/bjc.1994.194

**Published:** 1994-06

**Authors:** J. Kirk, J. Carmichael, I. J. Stratford, A. L. Harris

**Affiliations:** ICRF Laboratories, Institute of Molecular Medicine, Headington, UK.

## Abstract

The sensitivity of human breast and lung cancer cell lines to TGF-alpha-PE40, a novel chimeric recombinant cytotoxin composed of two independent domains, (i) TGF-alpha and (ii) a 40 kDa segment of the Pseudomonas exotoxin protein, PE-40, was investigated. Toxicity varied widely, correlated with epidermal growth factor receptor (EGFR) levels (P = 0.01) and was greatly reduced by EGF, indicating that binding of TGF-alpha-PE40 to EGFR is important in mediating toxicity. Cell lines expressing low EGFR levels were most highly protected by EGF, indicating that normal (low EGFR-expressing) tissue may be selectively protected by EGF in vivo. P-glycoprotein did not confer resistance to TGF-alpha-PE40, and toxicity was unaffected by multidrug resistance-modulating agents (cyclosporin A, tamoxifen, verapamil), indicating a role for TGF-alpha-PE40 in the clinical management of drug-resistant tumours.


Br. J. Cancer (1994), 69, 988–994 © Macmillan Press Ltd., 1994

### Selective toxicity of TGF-α–PE40 to EGFR-positive cell lines: selective protection of low EGFR-expressing cell lines by EGF

J. Kirk[1], J. Carmichael[2], I.J. Stratford[3] & A.L. Harris[2]

[1]*ICRF Laboratories, Institute of Molecular Medicine, Headington, OX3 9DU, UK;* [2]*ICRF Clinical Oncology Unit, Churchill Hospital, Headington, OX3 7LJ, UK;* [3]*MRC Radiobiology Unit, Didcot, OX11 0RD, UK.*

**Summary** The sensitivity of human breast and lung cancer cell lines to TGF-α–PE40, a novel chimeric recombinant cytotoxin composed of two independent domains, (i) TGF-α and (ii) a 40 kDa segment of the *Pseudomonas* exotoxin protein, PE-40, was investigated. Toxicity varied widely, correlated with epidermal growth factor receptor (EGFR) levels ($P = 0.01$) and was greatly reduced by EGF, indicating that binding of TGF-α–PE40 to EGFR is important in mediating toxicity. Cell lines expressing low EGFR levels were most highly protected by EGF, indicating that normal (low EGFR-expressing) tissue may be selectively protected by EGF *in vivo*. P-glycoprotein did not confer resistance to TGF-α–PE40, and toxicity was unaffected by multidrug resistance-modulating agents (cyclosporin A, tamoxifen, verapamil), indicating a role for TGF-α–PE40 in the clinical management of drug-resistant tumours.

Expression of epidermal growth factor receptors (EGFRs) has been detected in several malignant tumour types, including breast (Nicholson *et al.*, 1991), ovarian (Kohler *et al.*, 1992), lung and glial tumours (Chaffenet *et al.*, 1992; Fleming *et al.*, 1992). EGFR overexpression correlates with poor cellular differentiation (Bolla *et al.*, 1992), which is in turn a marker for poor prognosis in lymph node-negative breast cancer (Nicholson *et al.*, 1991), non-small-cell lung cancer (Veale *et al.*, 1993) and superficial bladder cancer (Smith *et al.*, 1989; Neal *et al.*, 1990). In breast cancer cells, increased expression correlates with reduced oestrogen receptor (ER) levels (Bilous *et al.*, 1992), and such an increase is associated with tamoxifen resistance (Nicholson *et al.*, 1989). Overexpression of EGFR is also associated with the multidrug resistance (MDR) phenotype (Shin *et al.*, 1991). There is therefore potential to exploit the difference between EGFR expression in normal and MDR-positive, malignant cells in order to target treatment to tumours which may respond poorly to chemotherapy (Harris, 1990).

We have assessed the toxicity of a chimeric cytotoxin composed of two independent domains: (i) transforming growth factor α (TGF-α) and (ii) PE40, a 40 kDa segment of *Pseudomonas* exotoxin (PE). The toxicity of multidomain PE is abolished when the cell targeting moiety is removed to yield PE40, which can neither bind to nor enter cells (Pastan & FitzGerald, 1989). Conjugating TGF-α to PE40 restores cell-binding activity, allowing selective entry of TGF-α–PE40 (TP40) into EGFR-positive cells. Normal tissue expressing low levels of EGFR may therefore be less susceptible to toxicity. We have tested this hypothesis by determining the toxicity of TP40 to human breast cancer and lung cancer cell lines expressing different levels of EGFR. P-glycoprotein (Pgp)-positive cell lines were also included in the study to determine whether MDR confers cross-resistance to the chimeric toxin. The effects of the MDR modifiers tamoxifen (Ramu *et al.*, 1984; Kirk *et al.*, 1993*a*,*b*), verapamil (Tsuruo *et al.*, 1981) and cyclosporin A (Twentyman *et al.*, 1987) on TP40 toxicity were also investigated; previous studies have suggested that verapamil may enhance the toxicity of chimeric toxins by altering PE40 processing in endosomes (Lyall *et al.*, 1987; Jaffrézou & Laurent, 1993).

### Materials and methods

### *Cell lines and tissue culture*

The panel of cell lines used comprised eight human breast cancer, four small-cell lung cancer (SCLC) and nine non-small-cell lung cancer (NSCLC) cell lines (Table I), which express EGFR at different levels. These included the breast cancer cell line MDA-468, which greatly overexpresses EGFR (Filmus *et al.*, 1985), and non-adherent SCLC cell lines, which do not express EGFR. The cell lines varied in sensitivity to cytotoxic drugs and included two MDR-positive cell lines expressing Pgp: (i) the *mdr*1 transfectant, S1/1.1, which has *mdr*1 levels at least 100-fold higher than its wild-type parental cell line, S1 (Baas *et al.*, 1990; F. Baas, personal communication); and (ii) MCF-7$^{Adr}$ (Batist *et al.*, 1986), derived from the human breast cancer cell line MCF-7 (Soule *et al.*, 1973) by chronic exposure to adriamycin. Two strains of MCF-7 cells were used: an early-passage strain (MCF-7$^{EP}$; passage 50–60) and a late-passage strain (MCF-7$^{LP}$; passage >300) in order to monitor changes in receptor status and drug sensitivity during long-term growth *in vitro*. S1 and S1/1.1 cells were kindly provided by F. Baas (University of Amsterdam, The Netherlands), other lung cancer cell lines by A.F. Gazdar (NCI Navy Medical Oncology Branch, Bethesda, MD, USA) and MCF-7$^{Adr}$ breast cancer cell lines by K. Cowan (NCI Clinical Pharmacology Branch).

All cell lines were maintained as monolayer cultures in Ham's F12 medium (S1 and S1/1.1) or RPMI-1640 medium (all other cell lines), each supplemented with 10% fetal calf serum (FCS) and 2 mM glutamine. Cultures were grown in 5% carbon dioxide under 100% humidity at 37°C and maintained in exponential growth phase by passaging twice weekly. All cell lines were regularly shown to be *Mycoplasma* free, and are listed in Table I.

### *Drugs*

TP40 (provided by David Heimbrook, Merck Sharpe & Dohme Research Laboratories, West Point, PA, USA), was diluted to 2 or 20 μM in phosphate-buffered solution (PBS), divided into aliquots and stored at −20°C. Tamoxifen, provided by ICI Pharmaceuticals (Macclesfield, UK), was prepared as a 50 mM stock solution in ethanol and stored at 4°C. Verapamil (Sigma, Dorset, UK) and cyclosporin A (Sandoz) were dissolved in dimethylsulphoxide (DMSO) to 20 mM and diluted in PBS as appropriate. EGF (tissue culture grade, from Sigma) was dissolved in PBS to 10 μg $ml^{-1}$, filtered (0.22 μm), aliquoted and stored at −20°C. Tamoxifen, verapamil and cyclosporin A were added to cells at their maximum non-toxic concentration (MNC), defined as the maximum concentration tested which reduced control cell optical density by less than 5% after 4 days' growth. Organic solvent levels did not exceed 0.1% by volume of the cell suspension, a concentration of vehicle demonstrated not to affect cell growth.

Correspondence: J. Kirk, University Laboratory of Physiology, University of Oxford, Parks Road, OX1 3PT, UK.
Received 12 October 1993.

**Table I** Characteristics of cell lines. Oestrogen and EGF receptor levels were determined from cytosol and membrane fractions respectively, derived from the same sample of cells. TP40 $IC_{50}$ values were determined by growing cells continuously for 4 days in the presence and absence of TP40 at a range of concentrations. Cell viability was assessed using a semiautomated MTT assay. TP40 $IC_{50}$ values are mean results from four separate determinations ± s.e.m.

| *Cell type* | *Cell line* | *ER*[a] | *EGFR*[a] | *TP40 $IC_{50}$ (pM)* |
|---|---|---|---|---|
| Breast cancer | MCF-7[EP] | 92 | 0 | 38 ± 4 |
| | MCF-7[LP] | 59 | 7 | 41 ± 8 |
| | MCF-7[Adr] | <5 | 405 | 152 ± 11 |
| | MDA-231 | <5 | 108 | 227 ± 25 |
| | MDA-361 | 60 | 5 | 649 ± 9 |
| | MDA-468 | <5 | 4,480 | 22 ± 3 |
| | SKBr3 | <5 | 674 | 116 ± 6 |
| | T47D | 47 | 52 | 74 ± 15 |
| | ZR75 | 14 | 120 | 132 ± 39 |
| SCLC | NCI-H 249 | <5 | 0 | 180,000 ± 6,300 |
| | NCI-N 417 | <5 | 32 | 2,360 ± 390 |
| | NCI-H 526 | 10 | 0 | 117,000 ± 9,900 |
| | NCI-H 841 | <5 | 723 | 43 ± 4 |
| NSCLC | NCI-H 226 | <5 | 632 | 46 ± 12 |
| | NCI-H 322 | <5 | 575 | 19 ± 3 |
| | NCI-H 358 | <5 | 184 | 36 ± 9 |
| | NCI-H 460 | <5 | 0 | 193 ± 24 |
| | NCI-H 522 | <5 | 10 | 35,100 ± 1,200 |
| | NCI-H 647 | <5 | 70 | 126 ± 17 |
| | S1 | <5 | 720 | 133 ± 16 |
| | S1/1.1 | <5 | 470 | 145 ± 15 |
| | A549 | <5 | 566 | 14 ± 2 |

[a]Cellular EGFR and ER levels are expressed in units of ligand bound (fmol $mg^{-1}$ protein).

*Cytotoxicity assays*

Exponentially growing cells were trypsinised, centrifuged and resuspended in fresh medium (10% FCS, 2 mM glutamine) at the appropriate cell density. Cell suspension (180 μl) was aliquoted into 96 well microtitre plates at a seeding density previously demonstrated to allow exponential growth for 4 days. Modifiers, drug (at eight different concentrations) and/or vehicle (10 μl) were added in quadruplicate at appropriate concentrations. Cells were incubated continuously with drug and/or modifier at 37°C (5% carbon dioxide, 100% humidity) for 4 days. Cytotoxicity was determined using the MTT [3-(4,5-dimethylthiazol-2-yl)-2,5-diphenyltetrazolium bromide] assay (Mosmann, 1983; Carmichael *et al.*, 1987). Aliquots of MTT (50 μl, 2 mg $ml^{-1}$) were dispensed into all wells and the cells incubated for a further 4 h. Plates were inverted to discard medium and formazan crystals were solubilised in 100 μl of DMSO with 25 μl of glycine buffer (0.1 M glycine/0.1 M sodium chloride, pH 10.5; Plumb *et al.*, 1989). Plates were agitated for 5 min, and optical densities determined immediately at 540 nm using a Titertek Multiskan Plus MKII ELISA plate reader. Data were stored and processed on a Macintosh SE/30 microcomputer.

*Receptor assays*

Cells (~$10^8$) in exponential growth phase, harvested by trypsinisation and washed three times with PBS, were resuspended in 4 ml of ice-cold homogenisation buffer (20 mM HEPES, 1.5 mM EDTA, 0.5 mM PMSF, 1 mM benzamidine, 10 ng $ml^{-1}$ ovomucoid, pH 7.4) and allowed to stand on ice for 30 min. Cells were lysed by homogenisation (30 strokes in a glass–glass homogeniser) on ice, centrifuged (3,000 r.p.m., 10 min, 4°C) to pellet nuclei and unbroken cells, and the resulting supernatants centrifuged at 100,000 ***g*** for 45 min at 4°C. Supernatants (cytosolic extracts) were removed and supplemented with ice-cold dithiothreitol (200 mM) to a final concentration of 2 mM. Membrane pellets were rinsed twice with 0.5 ml of ice-cold membrane resuspension buffer (50 mM Tris, 150 mM sodium chloride, pH 7.4) then resuspended by gentle pipetting in 1.5 ml of membrane resuspension buffer. Cytosolic ER levels were determined using an Abbott ER-EIA Monoclonal kit (Abbott Laboratories, North Chicago, USA) by Atilla Turkes (Tenovus Insitute for Cancer Research, University of Wales College of Medicine, Cardiff, UK). Membrane EGFR levels were determined by ligand binding using [$^{125}$I]EGF, as previously described (Nicholson *et al.*, 1988). ER and EGFR receptor levels are expressed in terms of ligand bound per unit cytosolic or membrane protein (fmol $mg^{-1}$) respectively, and the limit of sensitivity of the EGFR assay was 1 fmol $mg^{-1}$ of membrane protein.

*Data analysis*

Dose–response data were fitted to a four parameter equation using DeltaSoft ELISA Analysis software (BioMetallics, Princeton, NJ, USA). $IC_{50}$ values (concentration of drug causing a 50% reduction in control cell optical density) were determined by interpolating into the equation a value 50% of control cell optical density. $IC_{50}$ values are presented as the mean from at least four separate experiments ± the standard error of the mean (s.e.m.). Modification of drug toxicity is expressed as a modification factor (MF), calculated by dividing the $IC_{50}$ value determined in the presence of modifier by that measured in its absence. MF values $>1$ indicate protection from drug toxicity and values $<1$ enhancement of drug toxicity, while a value of 1 indicates no modification. TP40 $IC_{50}$ values determined in the presence and absence of modifiers were compared using Student's paired two-tail *t*-test. Correlations between EGFR and ER levels, and between cellular EGFR levels and sensitivity to TP40, were analysed by calculating Kendall's coefficient of rank correlation ($\tau$); significance levels (*P*, one-tailed test) determined from $\tau$ are quoted in the text.

### Results

*EGFR and ER levels*

EGFR levels ranged from 0 to 4,480 fmol $mg^{-1}$ (Table I). For breast cancer cell lines, an inverse relationship ($P = 0.01$)

was observed between EGFR and ER levels (Figure 1). MCF-$7^{LP}$ cells expressed lower ER levels and higher EGFR levels than MCF-$7^{EP}$, although both cell lines were highly ER positive. For SCLC cell lines, anchorage-independent NCI-H 249 and NCI-H 526 cells expressed no detectable EGFR, adherent NCI-H 841 cells expressed high EGFR levels (723 fmol $mg^{-1}$) and semiadherent NCI-N 417 cells expressed intermediate levels (32 fmol $mg^{-1}$), indicating a correlation between loss of anchorage independence and EGFR expression. NSCLC cell lines, with the exception of NCI-H 460 and NCI-H 522 cells, expressed high levels of EGFR.

*Effects of EGF on growth of lung and breast cancer cell lines*

Marked variability was observed between the effects of EGF (0.1–300 ng $ml^{-1}$) on the growth of breast (Figure 2a) and lung cancer cell lines (Figure 2b). Growth of highly EGFR-positive MDA-468 cells was inhibited 54% by 10 ng $ml^{-1}$ EGF, while 100 ng $ml^{-1}$ EGF stimulated MCF-$7^{Adr}$ cell growth by 22%. Stimulation of S1, S1/1.1 (data not shown) and NCI-H 226 cell growth occurred at 30–100 ng $ml^{-1}$ EGF. These cell lines expressed relatively high levels of EGFR (~350–500 fmol $mg^{-1}$), however NCI-H 322 and NCI-H 841, which also expressed high levels (575 and 723 fmol $mg^{-1}$ respectively), were unaffected by EGF treatment. No effect on other lung cancer cell lines that expressed low levels of EGFR (data not shown) was observed.

*TP40 toxicity*

TP40 was toxic to all cell lines tested, with a 13,000-fold range in $IC_{50}$ values observed (Table I). NCI-H 249, NCI-H 522 and NCI-H 526 cells, which expressed low levels of EGFR (0–10 fmol $mg^{-1}$), were most resistant to TP40 ($IC_{50}$ = 35,000–180,000 pM), while MDA-468 cells, which expressed the highest level detected (4,480 fmol $mg^{-1}$), were highly sensitive ($IC_{50}$ = 22 pM). Similarly, NCI-H 226, NCI-H 322, NCI-H 841 and A549 cells, which were also highly EGFR positive (566–723 fmol $mg^{-1}$), were very sensitive to TP40, with $IC_{50}$ values of 14–46 pM. This trend is illustrated in Figure 3, which shows TP40 $IC_{50}$ values plotted as a function of EGFR levels for the panel of breast and lung cancer cell lines. A correlation ($P = 0.01$) was observed, indicating an inverse relationship between TP40 $IC_{50}$ values and EGFR levels. This correlation was weakened by results obtained using NCI-H 460 and (early and late passage) MCF-7 cells which were sensitive to TP40 but expressed very low levels of EGFR (Table I), and masking these values gave a stronger correlation ($P = 0.005$).

Lung and breast cancer cell lines were also analysed separately, as these are two histologically and biologically distinct groups of cells. EGFR levels were related to TP40 sensitivity for the 13 lung cancer cell lines alone ($P = 0.005$). Weaker correlations were observed when this sample was divided into NSCLC ($P = 0.05$) and SCLC cell lines ($P = 0.05$), presumably because of the small sample number in each subgroup. No correlation was observed for breast cancer cell lines, even when values for MCF-7 cells were omitted.

S1 cells and their *mdr*1-transfected subline, S1/1.1, were equally sensitive to TP40, indicating that Pgp does not confer resistance to this toxin. MCF-$7^{Adr}$ cells were relatively sensitive to TP40 when compared with other cell lines tested, but more resistant than MCF-7 cells. However, this result probably reflects the unusual sensitivity of MCF-7 cells to TP40.

**Figure 1** Correlation between ER levels and EGFR levels for breast cancer cell lines. Values were determined from cytosolic and membrane fractions, respectively, prepared from the same sample of cells.

**Figure 2** Effects of EGF on growth of (**a**) breast cancer and (**b**) lung cancer cell lines. **a**, MCF-7 (△), MCF-7Adr (○), MDA-231 (●), MDA-468 (▲). **b**, S1 (▲), NCI-H 226 (○), NCI-H 322 (●), NCI-H 841 (△). Results are from one representative experiment ± s.d. calculated from four replicates. Where not shown, error bars fall within the area of plot symbols.

**Figure 3** TP40 $IC_{50}$ values plotted as a function of EGFR levels for (**a**) breast (●) and (**b**) non-small cell (▲) and small-cell (△) lung cancer cell lines. TP40 $IC_{50}$ values are means ± s.e.m. determined from at least four identical experiments.

**Figure 4** Effect of 0 (●), 10 (○), 100 (▲) and 500 ng $ml^{-1}$ (△) EGF on TP40 toxicity to (**a**) S1 and (**b**) S1/1.1 cells. Results are from one representative experiment ± s.d. calculated from four replicates. Where not shown, error bars fall within the area of plot symbols.

*Effects of modifiers on TP40 toxicity*

Figure 4a and b shows the effects of 0, 10, 100 and 500 ng $ml^{-1}$ .EGF on TP40 toxicity to S1 and S1/1.1 cells. EGF caused a concentration-dependent rightward shift in the dose–response curves, indicating antagonism of TP40 toxicity. This protective effect was observed with all cell lines tested, and $IC_{50}$ values for TP40 determined in the absence and presence of the highest concentration of EGF used (500 ng $ml^{-1}$) are listed in Table II. TP40 toxicity was reduced 81- (NCI-H 226) to 240-fold (S1/1.1). The degree of protection (MF) at 10 ng $ml^{-1}$ (Figure 5a) and 100 ng $ml^{-1}$ EGF (Figure 5b) was plotted as a function of EGFR levels for eight lung and breast cancer cell lines, and strong correlations ($r = -0.8$ and $-0.9$ respectively) were observed at each concentration, while the degree of protection at 500 ng $ml^{-1}$ EGF did not correlate with EGFR levels ($r = 0.01$, data not shown). At 10 and 100 ng $ml^{-1}$ EGF, therefore, cell lines expressing low levels (<50 fmol $mg^{-1}$) of EGFR were most highly protected from TP40 toxicity.

The MDR-modifying agents tamoxifen, verapamil and cyclosporin A did not alter TP40 toxicity to any cell line tested (Table II), although verapamil has previously been shown to enhance toxicity of a different PE conjugate, EGF-PE, to human KB cells (Lyall *et al.*, 1987; Jaffrézou & Laurent, 1993).

### Discussion

An inverse relationship was observed between ER and EGFR levels for the breast cancer cell lines, similar to results on human tumour biopsies previously published (Sainsbury *et al.*, 1985; Harris & Nicholson, 1988; Bilous *et al.*, 1992; Bolla *et al.*, 1992). EGFR-positive cells may have a growth advantage over hormone-dependent, EGFR-negative cells and outgrow ER-positive cells both *in vitro* and *in vivo*. This effect is apparent with wild-type MCF-7 cells, early-passage cells being EGFR-negative and highly ER-positive, while later passage cells express low levels of EGFR and reduced levels of ER. SCLC cell lines generally grow in an anchorage-independent manner and do not express EGFR, however loss of the non-adherent phenotype may occur during continuous growth *in vitro*. Such loss was correlated in our panel of cell lines with increased expression of EGFR. Gamou *et al.*

**Table II** Effects of tamoxifen, cyclosporin A, verapamil and EGF on TP40 toxicity to human breast and lung cancer cell lines. Cells were exposed to TP40 and/or modifiers continuously for 4 days. Cell viability was assessed using a semiautomated MTT assay. Results are mean values from at least five identical experiments ± s.e.m.

| | | | *TP40 $IC_{50}$ value (pM)* | | |
|---|---|---|---|---|---|
| *Cell line* | *+ PBS* | *+ EGF*[a] | *+ Tamoxifen*[b] | *+ CsA*[c] | *+ Verapamil*[c] |
| MCF-7[EP] | 38 ± 4 | 4,900 ± 1,300* | 32 ± 3 | 38 ± 8 | 34 ± 6 |
| *MF* | – | *130* | *1*[d] | *1* | *1* |
| MCF-7[Adr] | 152 ± 11 | 20,900 ± 800*** | 123 ± 21 | 192 ± 34 | 170 ± 31 |
| *MF* | – | *140* | *1* | *1* | *1* |
| MDA-468 | 22 ± 4 | – | 27 ± 1 | 29 ± 5 | 22 ± 9 |
| *MF* | – | – | *1* | *1* | *1* |
| S1 | 133 ± 16 | 25,600 ± 3,600** | 135 ± 17 | 132 ± 27 | 142 ± 19 |
| *MF* | – | *192* | *1* | *1* | *1* |
| S1/1.1 | 145 ± 16 | 34,400 ± 3,900** | 144 ± 13 | 121 ± 23 | 153 ± 38 |
| *MF* | – | *240* | *1* | *1* | *1* |
| NCI-H 226 | 46 ± 12 | 3,700 ± 900* | 47 ± 7 | 44 ± 8 | 41 ± 11 |
| *MF* | – | *81* | *1* | *1* | *1* |

[a]EGF was added to cells at 500 ng $ml^{-1}$. [b]Tamoxifen was added to cells at 1 μM (MCF-7 cells) or 10 μM (remaining cell lines). [c]Cyclosporin A (CsA) and verapamil were added to cells at 5 μM. [d]Where MF = 1, $P$ = 0.05–1. *$P$ = 0.01–0.05, **$P$ = 0.001–0.01, ***$P$ = 0.0001–0.001.

**Figure 5** Modification of TP40 toxicity by (**a**) 10 and (**b**) 100 ng $ml^{-1}$ EGF as a function of EGFR levels. Modulation of TP40 toxicity is expressed as a *modification factor* (ratio of TP40 $IC_{50}$ values determined in the presence and absence of EGF). Results are mean values calculated from five identical experiments ± s.e.m.

(1990) also observed increased levels of EGFR and loss of anchorage independence in SCLC variants isolated from cultures treated either with a demethylating agent (5-azacytidine) or with a tumour-promoting agent (12-*O*-tetradecanoylphorbol-13-acetate).

EGF stimulated growth of NCI-H 226, S1, S1/1.1 and MCF-7[Adr] cells, all of which expressed high levels of EGFR (350–630 fmol $mg^{-1}$). However, other cell lines which bound similar levels of EGF were unaffected by EGF. By contrast, MDA-468 cells, which expressed the highest EGFR levels detected, were growth inhibited by EGF. Such inhibition has been described by Filmus *et al.* (1985), and also demonstrated with the highly EGFR-positive cell line, A431 (Gill & Lazar, 1981). Kaplan *et al.* (1990), working with MDA-468 cells, have suggested that inhibition is an artefact of *in vitro* systems; EGF strongly stimulates glucose metabolism in highly EGFR-positive cells, thereby depleting the medium of this sugar and leading to cell death through glucose starvation.

We have assessed TP40 toxicity to a panel of cell lines, which displayed a 13,000-fold range in sensitivity. Cell lines most resistant to TP40 expressed little or no EGFR, while those most sensitive generally expressed high levels of EGFR. This correlation between EGFR expression and TP40 sensitivity for cells which are highly resistant or highly sensitive to TP40 supports the hypothesis that TP40 enters cells via binding of the TGF-α moiety to EGFR. Additional evidence is provided by the dose-dependent protection from toxicity observed in the presence of EGF. EGF may protect cells from TP40 toxicity either directly, through competition for binding to EGFR, and/or by down-regulating EGFR expression. At 10 and 100 ng $ml^{-1}$ EGF, cellular expression of EGFR correlated inversely with the degree of protection from toxicity, indicating that cell lines expressing low levels of EGFR were most highly protected. For cells expressing little EGFR, these concentrations of EGF apparently reduced binding of TP40 to levels insufficient to cause toxicity. Higher levels of EGF would presumably be required to prevent binding of TP40 to cells with high EGFR levels. Normal tissue EGFR expression is 5–50 fmol $mg^{-1}$ in non-squamous tissues (Ozawa *et al.*, 1988); EGF may therefore selectively protect such tissues from the effects of TP40 *in vivo*, leaving highly EGFR-positive tumour cells relatively more sensitive. Potential therefore exists for further enhancing the therapeutic ratio.

The correlation between TP40 sensitivity and EGFR levels did not apply to all cell lines; for example, NCI-H 460 and MCF-7 (both early and late passage) cells were sensitive to TP40 but expressed low levels of EGFR. NCI-H 460 cells were, however, exquisitely sensitive to a wide range of cytotoxic drugs (e.g. adriamycin, AMSA, cisplatin, vinblastine and VP16; S. Houlbrook, unpublished data), yet were more resistant to TP40 than 14 of the 21 cell lines included in this study. NCI-H 460 cells could therefore be considered relatively resistant to TP40, when the extreme sensitivity to chemotherapeutic agents is taken into consideration. The inability to *perfectly* correlate EGFR levels with TP40 $IC_{50}$ values indicates that sensitivity to TP40 is not related solely to the ability of the toxin to enter cells, although this may be the predominant factor for cell lines with very high or very low EGFR levels. Sensitivity to TP40 could occur via multiple mechanisms, of which enhanced transport into the cell (via EGFR) is only one. After binding to the cell membrane, TP40 is internalised within endosomes, where in order to be released into the cytosol it must first be proteolytically processed to a 37 kDa fragment (PE37; Theuer *et al.*, 1992). Once in the cytosol, the catalytic domain of the toxin ADP-ribosylates elongation factor 2, thereby inhibiting protein synthesis. Theuer *et al.* (1992, 1993) found a TGF-α–PE37 conjugate to much more toxic than a derivative containing full-length PE, indicating that proteolytic processing is not 100% efficient, and may be rate limiting. Processing rates and efficiency may vary between different cell lines, perhaps explaining different sensitivities to TP40 of cell lines with similar EGFR levels.

Although TP40 was less toxic to Pgp-positive MCF-$7^{Adr}$ cells than to wild-type MCF-7 cells, S1 and S1/1.1 cells were equally sensitive to TP40, and toxicity was not altered by the MDR modulators tamoxifen, cyclosporin A or verapamil. It is therefore unlikely that Pgp confers resistance to TP40, and this toxin may be useful in the treatment of drug-resistant tumours. Lyall *et al.* (1987) found that verapamil enhanced toxicity of another PE conjugate, EGF–PE, to human KB cells, and indeed many MDR modulators have also been shown to enhance toxicity of ligand–toxin conjugates (Jaffrézou & Laurent, 1993). Verapamil may sensitise cells to PE conjugates by altering the pH within endosomes, thereby enhancing translocation of the toxin to the cytosol (Lyall *et al.*, 1987). However, Jaffrézou and Laurent (1993) suggest that a common mechanism, such as altered lipid metabolism, may account for enhanced toxicity of both ligand–toxin conjugates and drugs associated with MDR. Such sensitisation was not observed in the present study, indicating that this effect may be specific either to the cell type or to the PE conjugate used.

In conclusion, the recombinant cytotoxic protein TP40 was highly toxic to EGFR-positive cells at picomolar concentrations, while EGFR-negative cell lines tended to be more resistant. Tumour cells refractory to conventional chemotherapy frequently overexpress EGFR (Smith *et al.*, 1989; Nicholson *et al.*, 1991) and may therefore be sensitive to treatment with TP40. Low EGFR-expressing cells were selectively protected from TP40 toxicity by EGF (10–100 ng $ml^{-1}$), and EGF may have a role in combination with TP40 to target highly EGFR-positive tumours *in vivo*. Expression of Pgp was not associated with resistance to TP40, which may also be effective in the treatment of MDR-positive tumours.

This work was supported by the Imperial Cancer Research Fund and performed at the MRC Radiobiology Unit (Chilton, Didcot, UK) and the Institute of Molecular Medicine (Headington, Oxford).

**Abbreviations:** EGF, epidermal growth factor; EGFR, epidermal growth factor receptor; ER, oestrogen receptor; FCS, fetal calf serum; MDR, multidrug resistance; PE, *Pseudomonas* exotoxin; PE40, 40 kDa unit of *Pseudomonas* exotoxin; Pgp, P-glycoprotein; TGF-α, transforming growth factor α; TP40, TGF-α–PE40.

### References

BAAS, R., JONGSMA, A.P.M., BROXTERMAN, H.J., ARCECI, R.J., HOUSMAN, D., SCHEFFER, G.L., RIETHORST, A., VAN GROENIGEN, M., NIEUWINT, A.W.M. & JOENJE, H. (1990). Non-P-glycoprotein mediated mechanism for multidrug resistance precedes P-glycoprotein expression during *in vitro* selection for doxorubicin resistance in a human lung cancer cell line. *Cancer Res.*, **50**, 5392–5398.

BATIST, G., TULPULE, A., SINHA, B.K., KATKI, A.G., MYERS, C.E. & COWAN, K.H. (1986). Overexpression of a novel anionic glutathione transferase in multidrug-resistant human breast cancer cells. *J. Biol. Chem.*, **261**, 15544–15549.

BILOUS, M., MILLIKEN, J. & MATHIJS, J.-M. (1992). Immunocytochemistry and *in situ* hybridisation of epidermal growth factor receptor and relation to prognostic factors in breast cancer. *Eur. J. Cancer*, **28A**, 1033–1037.

BOLLA, M., CHEDIN, M., COLONNA, M., MARRON, J., ROSTAING-PUISSANT, B. & CHAMBAZ, E. (1992). Prognostic value of epidermal growth factor receptor in a series of 303 breast cancers. *Eur. J. Cancer*, **28A**, 1052–1054.

CARMICHAEL, J., DEGRAFF, W.G., GAZDAR, A.F., MINNA, J.D. & MITCHELL, J.B. (1987). Evaluation of a tetrazolium-based semi-automated colorimetric assay: assessment of chemosensitivity testing. *Cancer Res.*, **47**, 936–942.

CHAFFENET, M., CHAUVIN, C., LAINE, M., BERGER, F., CHEDIN, M., ROST, N., NISSOU, M. & BENABID, A.L. (1992). EGF receptor amplification and expression in human brain tumours. *Eur. J. Cancer*, **28**, 11–17.

FILMUS, J., POLLACK, M.N., CAILLEAU, R. & BUICK, R.N. (1985). MDA-468, a human breast cancer cell line with a high number of epidermal growth factor (EGF) receptors, has an amplified EGF receptor gene and is growth inhibited by EGF. *Biochem. Biophys. Res. Commun.*, **128**, 898–905.

FLEMING, T.P., SAXENA, A., CLARK, W.C., ROBERTSON, J.T., OLDFIELD, E.H., AARONSEN, S.A. & ALI, I.U. (1992). Amplification and/or overexpression of platelet-derived growth factor receptors and epidermal growth factor receptor in human glial tumors. *Cancer Res.*, **52**, 4550–4553.

GAMOU, S., SHIMOSATO, Y. & SHIMIZU, N. (1990). Regulation of the epidermal growth factor receptor gene expression in a morphological variant isolated from an epidermal growth factor receptor-deficient small cell lung carcinoma cell line. *Cell Growth Different.*, **1**, 351–359.

GILL, G.N. & LAZAR, C.S. (1981). Increased phosphotyrosine content and inhibition of proliferation in EGF-treated A431 cells. *Nature*, **293**, 305–307.

HARRIS, A.L. & NICHOLSON, S. (1988). Epidermal growth factor receptors in human breast cancer. In *Breast Cancer: Cellular and Molecular Biology*. Lippman, M.E. & Dickson, R. (eds), pp. 93–119. Kluwer: Boston.

HARRIS, A.L. (1990). The epidermal growth factor receptor as a target for therapy. *Cancer Cells*, **2**, 321–323.

JAFFREZOU, J.-P. & LAURENT, G. (1993). The intriguing link between modulation of both multidrug resistance and ligand-toxin conjugate cytotoxicity. *FEBS Lett.*, **323**, 191–197.

KAPLAN, O., JAROSZEWSKI, J.W., FAUSTINO, P.J., ZUGMAIER, G., ENNIS, B.W., LIPPMAN, M. & COHEN, J.S. (1990). Toxicity and effects of epidermal growth factor on glucose metabolism of MDA-468 human breast cancer cells. *J. Biol. Chem.*, **265**, 13641–13649.

KIRK, J., HOULBROOK, S., STUART, N.S.A., STRATFORD, I.J., HARRIS, A.L. & CARMICHAEL, J. (1993*a*). Selective reversal of vinblastine resistance in multidrug-resistant cell lines by tamoxifen, toremifene and their metabolites. *Eur. J. Cancer*, **29A**, 1152–1157.

KIRK, J., HOULBROOK, S., STUART, N.S.A., STRATFORD, I.J., HARRIS, A.L. & CARMICHAEL, J. (1993*b*). Differential modulation of doxorubicin toxicity to multidrug and intrinsically drug resistant cell lines by anti-oestrogens and their major metabolites. *Br. J. Cancer*, **67**, 1189–1195.

KOHLER, M., BAUKNECHT, T., GRIMM, M., BIRMELIN, G., KOMMOSS, F. & WAGNER, E. (1992). Epidermal growth factor receptor and transforming growth factor alpha expression in human ovarian carcinomas. *Eur. J. Cancer*, **28A**, 1432–1437.

LYALL, R.M., HWANG, J., CARDERELLI, C., FITZGERALD, D., AKIYAMA, S., GOTTESMAN, M. & PASTAN, I. (1987). Isolation of human KB cell lines resistant to epidermal growth factor–*Pseudomonas* exotoxin conjugates. *Cancer Res.*, **47**, 2961–2966.

MOSMANN, T. (1983). Rapid colorimetric assay for cellular growth and survival: application to proliferation in cytotoxicity assays. *J. Immunol. Methods*, **65**, 55–63.

NEAL, D.E., SHARPLES, L., SMITH, K., FENNELLY, J., HALL, R.R. & HARRIS, A.L. (1990). The epidermal growth factor receptor and the prognosis of bladder cancer. *Cancer*, **65**, 1619–1625.

NICHOLSON, S., SAINSBURY, J.R.C., NEEDHAM, G.K., CHAMBERS, P., FARNDON, J.R. & HARRIS, A.L. (1988). Quantitative assays of epidermal growth factor receptor in human breast cancer: cut-off points of clinical relevance. *Int. J. Cancer*, **42**, 36–41.

NICHOLSON, S., SAINSBURY, J.R.C., HALCROW, P., CHAMBERS, P., FARNDON, J.R. & HARRIS, A.L. (1989). Expression of epidermal growth factor receptors associated with lack of response to endocrine therapy in recurrent breast cancer. *Lancet*, **1**, 182–185.

NICHOLSON, S., RICHARD, J., SAINSBURY, J.R.C., HALCROW, P., KELLY, P., ANGUS, B., WRIGHT, C., HENRY, J., FARNDON, J.R. & HARRIS, A.L. (1991). Epidermal growth factor receptor: results of a 6 year follow-up study in operable breast cancer with emphasis on the node-negative subgroup. *Br. J. Cancer*, **63**, 146–150.

OZAWA, S., UEDA, M., ANDO, N., ABE, O. & SHIMIZU, N. (1988). Epidermal growth factor receptors in cancer tissues of esophagus, lung, pancreas, colorectum, breast and stomach. *Jpn J. Cancer Res.*, **79**, 1201–1207.

PASTAN, I. & FITZGERALD, D. (1989). *Pseudomonas* exotoxin: chimeric toxins. *J. Biol. Chem.*, **264**, 15157–15160.

PLUMB, J.A., MILROY, R. & KAYE, S.B. (1989). Effects of the pH dependence of 3-(4,5-dimethylthiazol-2-yl)2,5-diphenyltetrazolium bromide-formazan absorption on chemosensitivity determined by a novel tetrazolium-based assay. *Cancer Res.*, **49**, 4435–4440.

RAMU, A., GLAUBIGER, D. & FUKS, Z. (1984). Reversal of acquired resistance to doxorubicin in P388 murine leukemia cells by tamoxifen and other triparanol analogues. *Cancer Res.*, **44**, 4392–4395.

SAINSBURY, J.R.C., SHERBET, G.V., FARNDON, J.R. & HARRIS, A.L. (1985). Epidermal growth factor receptors and oestrogen receptors in human breast cancer. *Lancet*, **i**, 3654–3666.

SHIN, H.J., SHIN, D.M., GRANT, G., HONG, W.K. & PATHAK, S. (1991). Simultaneous amplification of epidermal growth factor-receptor and multidrug-resistance genes in a newly established human lung cancer cell line. *Anticancer Res.*, **11**, 241–248.

SMITH, K., FENNELLY, J.A., NEAL, D.E., HALL, R.R. & HARRIS, A.L. (1989). Characterization and quantitation of the epidermal growth factor receptor in invasive and superficial bladder tumors. *Cancer Res.*, **49**, 5810–5815.

SOULE, D., VAZQUEZ, J., LONG, A., ALBERT, S. & BRENNAN, M. (1973). A human cell line from a pleural effusion derived from a breast carcinoma. *J. Natl Cancer Inst.*, **51**, 1409–1413.

THEUER, C.P., FITZGERALD, D.J. & PASTAN, I. (1992). A recombinant form of *Pseudomonas* exotoxin directed at the epidermal growth factor receptor that is cytotoxic without requiring proteolytic processing. *J. Biol. Chem.*, **26**, 16872–16877.

THEUER, C.P., KREITMAN, R.J., FITZGERALD, D.J. & PASTAN, I. (1993). Immunotoxins made with a recombinant form of *Pseudomonas* exotoxin A that do not require proteolysis for activity. *Cancer Res.*, **53**, 340–347.

TSURUO, T., IIDA, H., TSUKAGOSHI, S. & SAKURAI, Y. (1981). Overcoming of vincristine resistance in P388 leukemia *in vivo* and *in vitro* through enhanced cytotoxicity of vincristine and vinblastine by verapamil. *Cancer Res.*, **41**, 1967–1972.

TWENTYMAN, P.R., FOX, N.E. & WHITE, D.J.G. (1987). Cyclosporin A and its analogues as modifiers of adriamycin and vincristine resistance in a multidrug resistant human lung cancer cell line. *Br. J. Cancer*, **56**, 55–57.

VEALE, D., KERR, N., GIBSON, G.H., KELLY, P.J. & HARRIS, A.L. (1993). The relationship of quantitative epidermal growth factor receptor expression in non-small cell lung cancer to long term survival. *Br. J. Cancer*, **68**, 162–165.


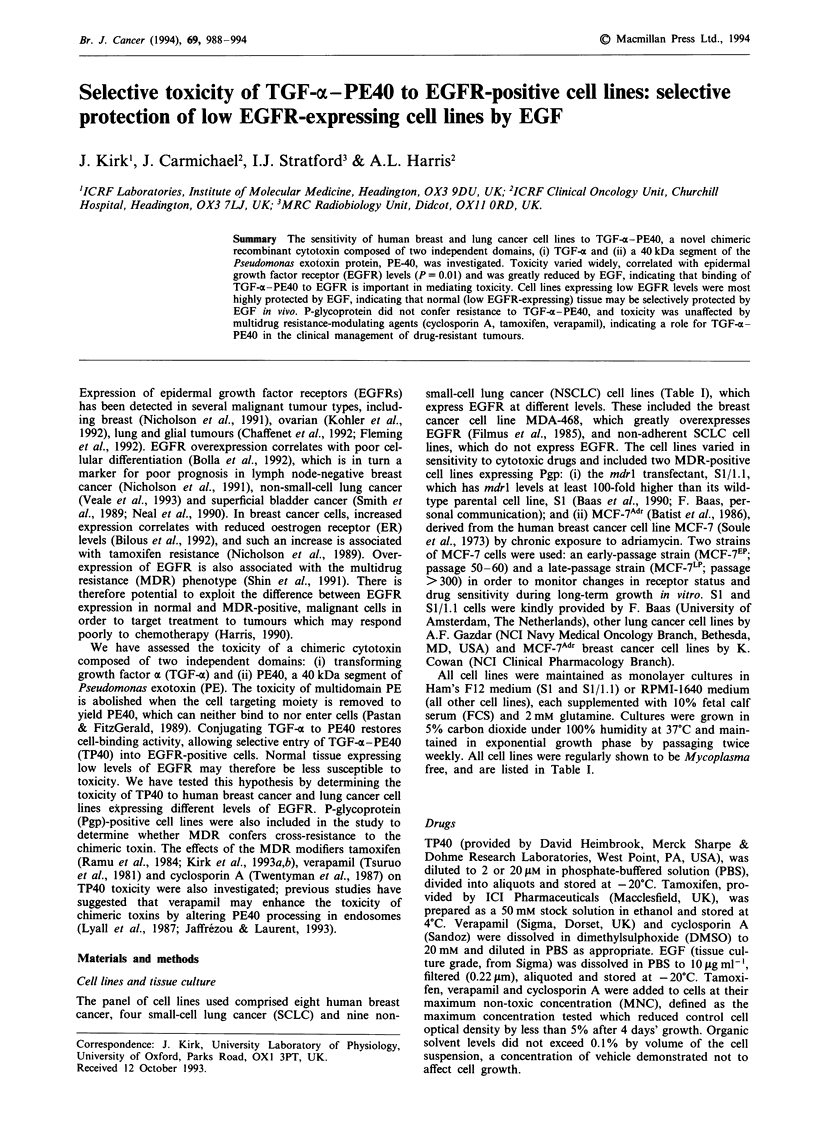

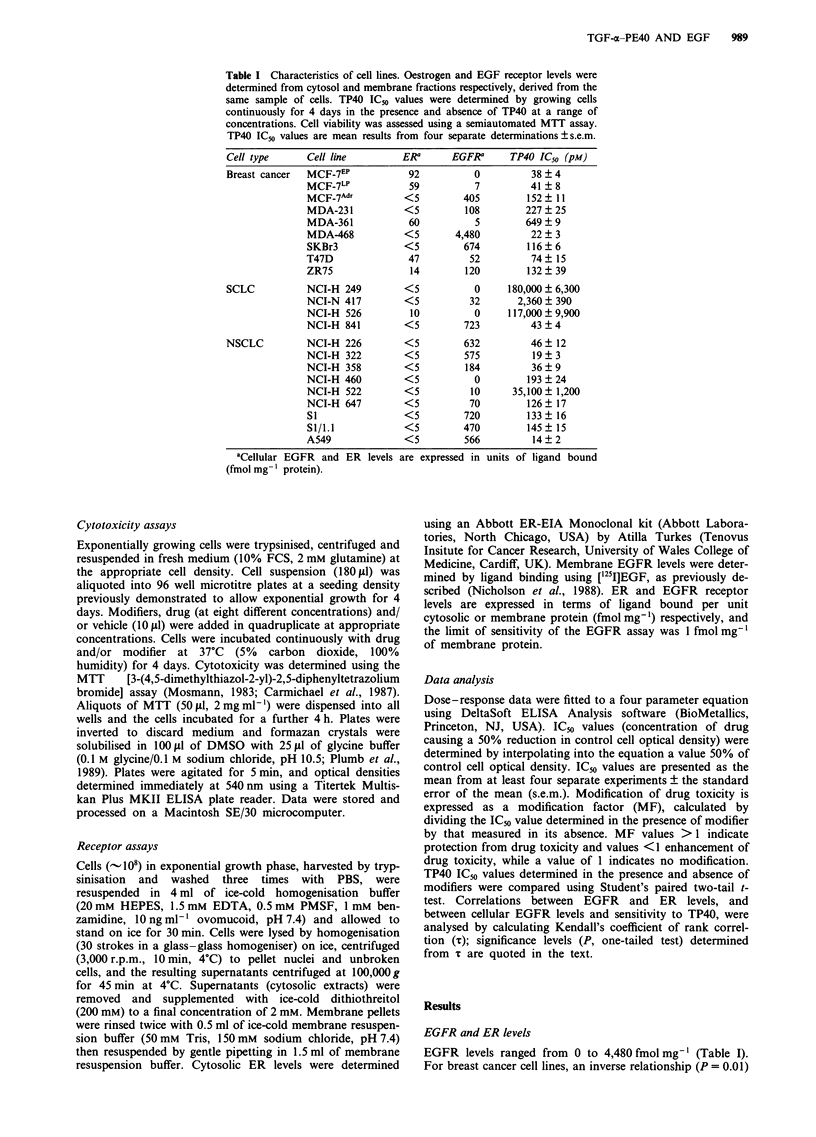

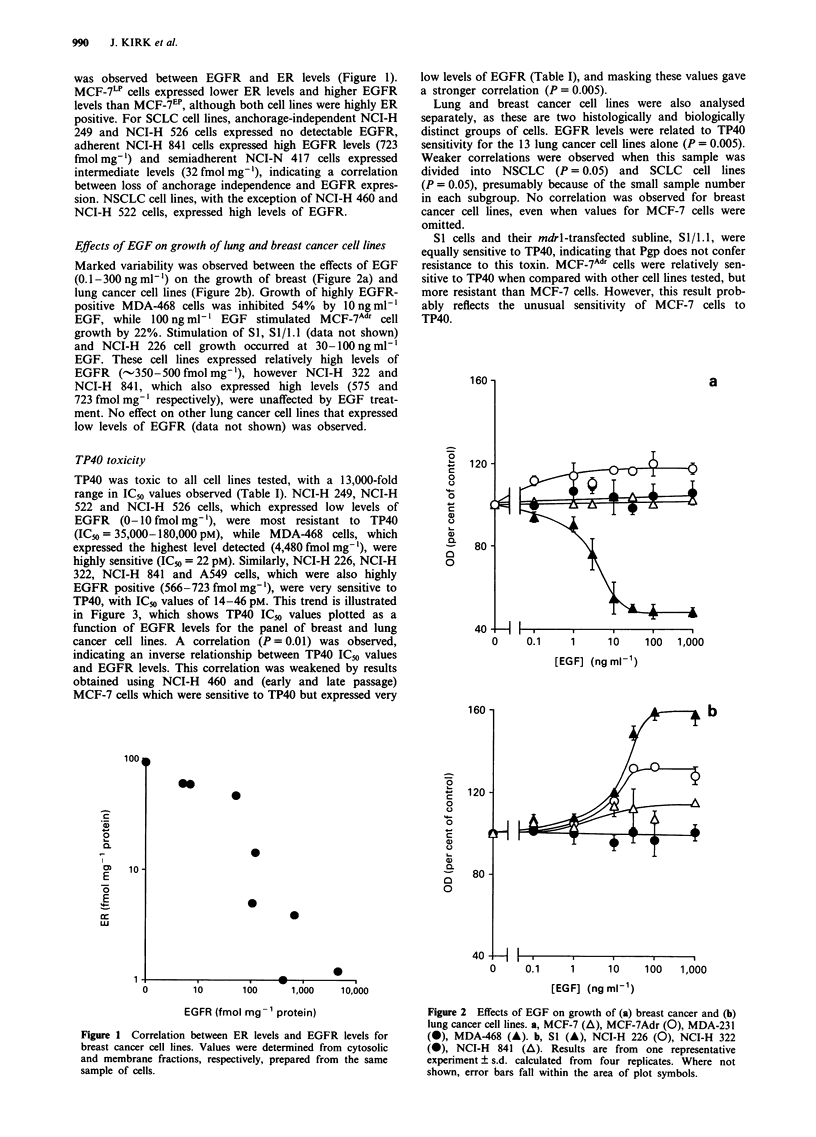

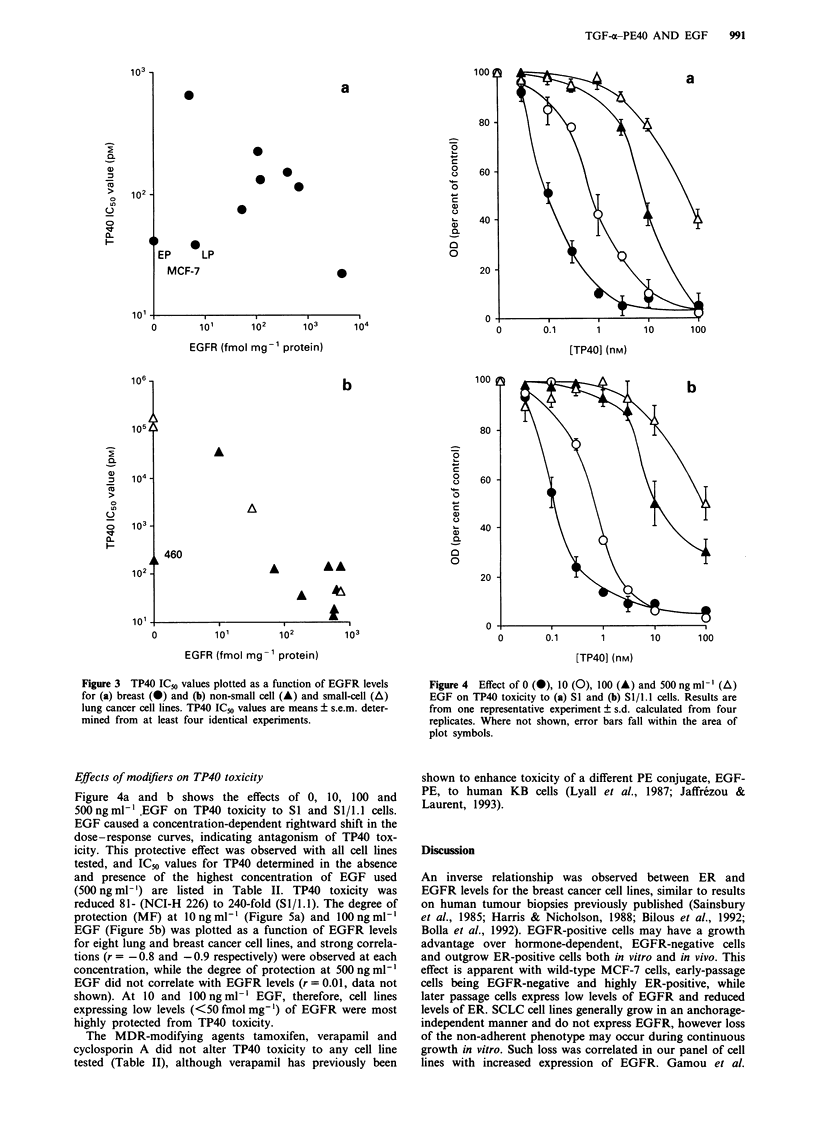

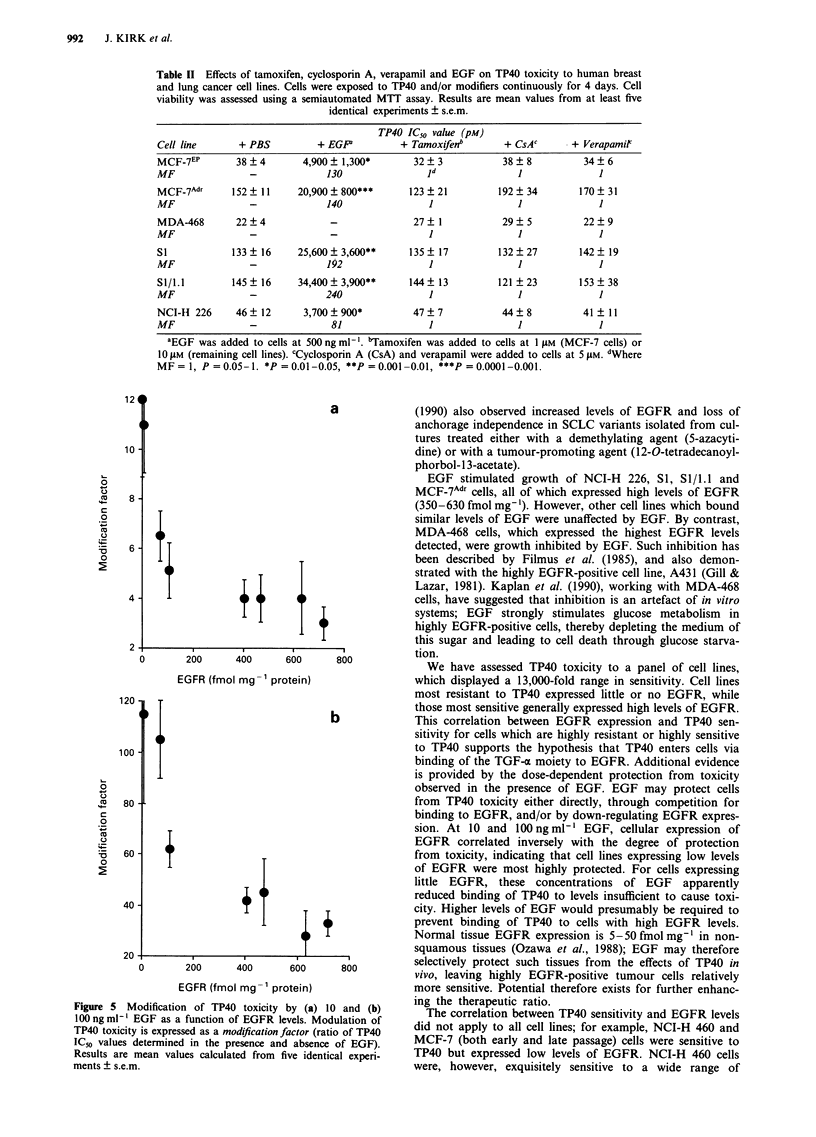

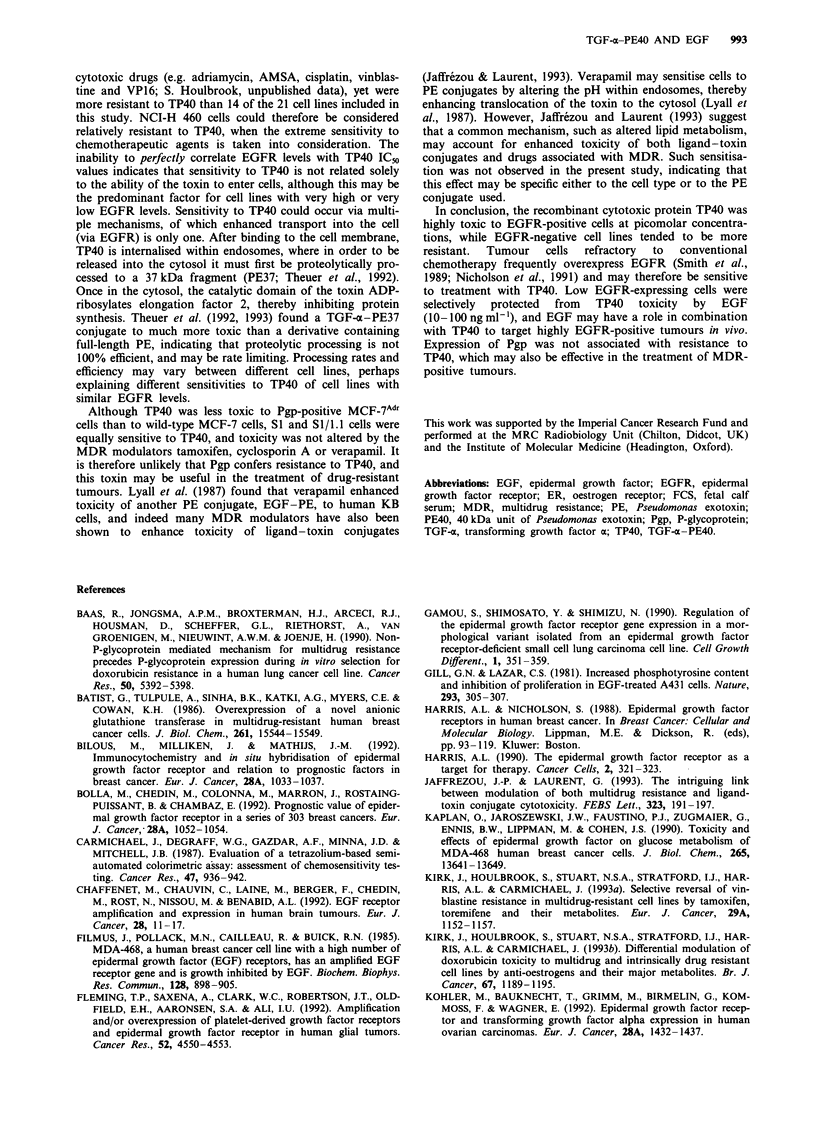

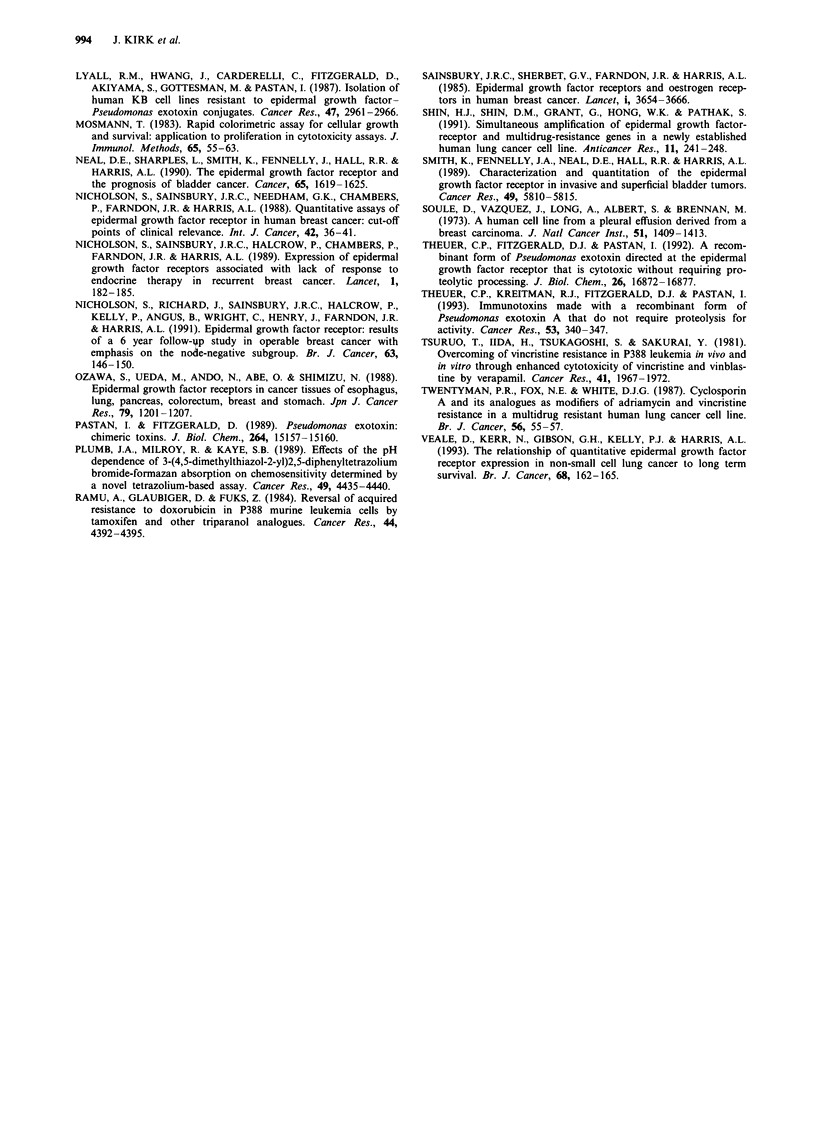


## References

[OCR_00864] Baas F., Jongsma A. P., Broxterman H. J., Arceci R. J., Housman D., Scheffer G. L., Riethorst A., van Groenigen M., Nieuwint A. W., Joenje H. (1990). Non-P-glycoprotein mediated mechanism for multidrug resistance precedes P-glycoprotein expression during in vitro selection for doxorubicin resistance in a human lung cancer cell line.. Cancer Res.

[OCR_00873] Batist G., Tulpule A., Sinha B. K., Katki A. G., Myers C. E., Cowan K. H. (1986). Overexpression of a novel anionic glutathione transferase in multidrug-resistant human breast cancer cells.. J Biol Chem.

[OCR_00879] Bilous M., Milliken J., Mathijs J. M. (1992). Immunocytochemistry and in situ hybridisation of epidermal growth factor receptor and relation to prognostic factors in breast cancer.. Eur J Cancer.

[OCR_00887] Bolla M., Chedin M., Colonna M., Marron J., Rostaing-Puissant B., Chambaz E. (1992). Prognostic value of epidermal growth factor receptor in a series of 303 breast cancers.. Eur J Cancer.

[OCR_00891] Carmichael J., DeGraff W. G., Gazdar A. F., Minna J. D., Mitchell J. B. (1987). Evaluation of a tetrazolium-based semiautomated colorimetric assay: assessment of chemosensitivity testing.. Cancer Res.

[OCR_00897] Chaffanet M., Chauvin C., Lainé M., Berger F., Chédin M., Rost N., Nissou M. F., Benabid A. L. (1992). EGF receptor amplification and expression in human brain tumours.. Eur J Cancer.

[OCR_00903] Filmus J., Pollak M. N., Cailleau R., Buick R. N. (1985). MDA-468, a human breast cancer cell line with a high number of epidermal growth factor (EGF) receptors, has an amplified EGF receptor gene and is growth inhibited by EGF.. Biochem Biophys Res Commun.

[OCR_00912] Fleming T. P., Saxena A., Clark W. C., Robertson J. T., Oldfield E. H., Aaronson S. A., Ali I. U. (1992). Amplification and/or overexpression of platelet-derived growth factor receptors and epidermal growth factor receptor in human glial tumors.. Cancer Res.

[OCR_00917] Gamou S., Shimosato Y., Shimizu N. (1990). Regulation of the epidermal growth factor receptor gene expression in a morphological variant isolated from an epidermal growth factor receptor-deficient small cell lung carcinoma cell line.. Cell Growth Differ.

[OCR_00924] Gill G. N., Lazar C. S. (1981). Increased phosphotyrosine content and inhibition of proliferation in EGF-treated A431 cells.. Nature.

[OCR_00935] Harris A. L. (1990). The epidermal growth factor receptor as a target for therapy.. Cancer Cells.

[OCR_00939] Jaffrézou J. P., Laurent G. (1993). The intriguing link between modulation of both multidrug resistance and ligand-toxin conjugate cytotoxicity.. FEBS Lett.

[OCR_00944] Kaplan O., Jaroszewski J. W., Faustino P. J., Zugmaier G., Ennis B. W., Lippman M., Cohen J. S. (1990). Toxicity and effects of epidermal growth factor on glucose metabolism of MDA-468 human breast cancer cells.. J Biol Chem.

[OCR_00960] Kirk J., Houlbrook S., Stuart N. S., Stratford I. J., Harris A. L., Carmichael J. (1993). Differential modulation of doxorubicin toxicity to multidrug and intrinsically drug resistant cell lines by anti-oestrogens and their major metabolites.. Br J Cancer.

[OCR_00953] Kirk J., Houlbrook S., Stuart N. S., Stratford I. J., Harris A. L., Carmichael J. (1993). Selective reversal of vinblastine resistance in multidrug-resistant cell lines by tamoxifen, toremifene and their metabolites.. Eur J Cancer.

[OCR_00967] Kohler M., Bauknecht T., Grimm M., Birmelin G., Kommoss F., Wagner E. (1992). Epidermal growth factor receptor and transforming growth factor alpha expression in human ovarian carcinomas.. Eur J Cancer.

[OCR_00973] Lyall R. M., Hwang J. L., Cardarelli C., FitzGerald D., Akiyama S., Gottesman M. M., Pastan I. (1987). Isolation of human KB cell lines resistant to epidermal growth factor-Pseudomonas exotoxin conjugates.. Cancer Res.

[OCR_00978] Mosmann T. (1983). Rapid colorimetric assay for cellular growth and survival: application to proliferation and cytotoxicity assays.. J Immunol Methods.

[OCR_00983] Neal D. E., Sharples L., Smith K., Fennelly J., Hall R. R., Harris A. L. (1990). The epidermal growth factor receptor and the prognosis of bladder cancer.. Cancer.

[OCR_01001] Nicholson S., Richard J., Sainsbury C., Halcrow P., Kelly P., Angus B., Wright C., Henry J., Farndon J. R., Harris A. L. (1991). Epidermal growth factor receptor (EGFr); results of a 6 year follow-up study in operable breast cancer with emphasis on the node negative subgroup.. Br J Cancer.

[OCR_00994] Nicholson S., Sainsbury J. R., Halcrow P., Chambers P., Farndon J. R., Harris A. L. (1989). Expression of epidermal growth factor receptors associated with lack of response to endocrine therapy in recurrent breast cancer.. Lancet.

[OCR_00988] Nicholson S., Sainsbury J. R., Needham G. K., Chambers P., Farndon J. R., Harris A. L. (1988). Quantitative assays of epidermal growth factor receptor in human breast cancer: cut-off points of clinical relevance.. Int J Cancer.

[OCR_01009] Ozawa S., Ueda M., Ando N., Abe O., Shimizu N. (1988). Epidermal growth factor receptors in cancer tissues of esophagus, lung, pancreas, colorectum, breast and stomach.. Jpn J Cancer Res.

[OCR_01015] Pastan I., FitzGerald D. (1989). Pseudomonas exotoxin: chimeric toxins.. J Biol Chem.

[OCR_01019] Plumb J. A., Milroy R., Kaye S. B. (1989). Effects of the pH dependence of 3-(4,5-dimethylthiazol-2-yl)-2,5-diphenyl-tetrazolium bromide-formazan absorption on chemosensitivity determined by a novel tetrazolium-based assay.. Cancer Res.

[OCR_01025] Ramu A., Glaubiger D., Fuks Z. (1984). Reversal of acquired resistance to doxorubicin in P388 murine leukemia cells by tamoxifen and other triparanol analogues.. Cancer Res.

[OCR_01036] Shin H. J., Shin D. M., Grant G., Hong W. K., Pathak S. (1991). Simultaneous amplification of epidermal growth factor-receptor and multidrug-resistance genes in a newly. Established human lung cancer cell line.. Anticancer Res.

[OCR_01042] Smith K., Fennelly J. A., Neal D. E., Hall R. R., Harris A. L. (1989). Characterization and quantitation of the epidermal growth factor receptor in invasive and superficial bladder tumors.. Cancer Res.

[OCR_01048] Soule H. D., Vazguez J., Long A., Albert S., Brennan M. (1973). A human cell line from a pleural effusion derived from a breast carcinoma.. J Natl Cancer Inst.

[OCR_01053] Theuer C. P., FitzGerald D., Pastan I. (1992). A recombinant form of Pseudomonas exotoxin directed at the epidermal growth factor receptor that is cytotoxic without requiring proteolytic processing.. J Biol Chem.

[OCR_01059] Theuer C. P., Kreitman R. J., FitzGerald D. J., Pastan I. (1993). Immunotoxins made with a recombinant form of Pseudomonas exotoxin A that do not require proteolysis for activity.. Cancer Res.

[OCR_01065] Tsuruo T., Iida H., Tsukagoshi S., Sakurai Y. (1981). Overcoming of vincristine resistance in P388 leukemia in vivo and in vitro through enhanced cytotoxicity of vincristine and vinblastine by verapamil.. Cancer Res.

[OCR_01071] Twentyman P. R., Fox N. E., White D. J. (1987). Cyclosporin A and its analogues as modifiers of adriamycin and vincristine resistance in a multi-drug resistant human lung cancer cell line.. Br J Cancer.

[OCR_01077] Veale D., Kerr N., Gibson G. J., Kelly P. J., Harris A. L. (1993). The relationship of quantitative epidermal growth factor receptor expression in non-small cell lung cancer to long term survival.. Br J Cancer.

